# The Mediating Role of Adiposity in the Association Between Respiratory Muscle Strength and Exercise Energy Expenditure in Adult Women: A Cross-Sectional Study

**DOI:** 10.3390/jcm15072629

**Published:** 2026-03-30

**Authors:** Monira I. Aldhahi, Daad Alhumaid, Dalia Binshaye, Fatimah Almohsen, Rand Alotaibi, Leen Bahathiq

**Affiliations:** Department of Rehabilitation Sciences, College of Health and Rehabilitation Sciences, Princess Nourah bint Abdulrahman University, Riyadh 11671, Saudi Arabia; 442002337@pnu.edu.sa (D.A.); 442005007@pnu.edu.sa (D.B.); 442001274@pnu.edu.sa (F.A.); 443000531@pnu.edu.sa (R.A.); 443000963@pnu.edu.sa (L.B.)

**Keywords:** breathing, physical activity, respiratory muscle strength, obesity, cardiorespiratory fitness

## Abstract

**Background and Objectives:** Obesity affects over 1.9 billion adults globally, with a disproportionately higher prevalence in Saudi Arabia among women. While excessive adiposity is known to impair respiratory mechanics and lung function, its relationship with respiratory muscle strength and exercise energy expenditure remains inadequately elucidated. This study examined differences in respiratory muscle strength, metabolic equivalents (METs) of physical activity, and energy expenditure during exercise between adults with normal and high body fat percentage (BF%) and explored the statistical role of body fat as a potential mediator in the cross-sectional association between respiratory muscle strength and energy expenditure. **Methods:** In this cross-sectional study, 126 Saudi women aged 18–45 years (mean age: 21.7 ± 4.2 years) were stratified into normal (*n* = 63) and high (*n* = 63) BF% groups. Body composition was assessed via bioelectrical impedance analysis, and respiratory muscle strength (MIP and MEP) was measured using a MicroRPM device. Peak oxygen consumption (VO_2peak_) and energy expenditure were obtained through the Bruce Submaximal Treadmill Protocol, and physical activity was self-reported via the IPAQ. Hierarchical regression and structural equation modeling were used to examine variable associations and explore statistical mediation patterns. **Results:** Participants with high body fat demonstrated significantly low MIP (−26%) and MEP (−31%), low VO_2peak_ (−13%), and approximately 26% high energy expenditure during exercise compared to the normal-BF group (all *p* < 0.001), despite comparable self-reported physical activity levels. Body fat percentage was the most strongly associated with energy expenditure (β = 0.078, R^2^ = 0.329), with maximal inspiratory pressure contributing an additional 7.3% of explained variance in hierarchical regression (total R^2^ = 0.414). Mediation analyses revealed that body fat percentage was statistically consistent with a partial mediation model in the relationship between MIP and energy expenditure (indirect association = −0.016, *p* = 0.033), accounting for 27% of the total association, and between MEP and energy expenditure (indirect association = −0.013, *p* = 0.035), accounting for 38% of the total association. **Conclusions:** High BF% is independently associated with low respiratory muscle strength and high exercise metabolic cost. Body fat is statistically associated with (and consistent with a mediating role in) an inverse relationship between respiratory muscle strength and energy expenditure. Alternative directional relationships and shared underlying factors may explain these observations.

## 1. Introduction

Body fat percentage (BF%) has emerged as a critical indicator of metabolic health, extending beyond conventional anthropometric measures, such as body mass index (BMI), to more precisely reflect adiposity-related physiological dysfunction [1]. Excessive accumulation of body fat is recognized as strongly associated with impaired cardiorespiratory function, altered energy metabolism, and physical performance [2]. Physical activity, defined by the World Health Organization as any bodily movement produced by skeletal muscles that requires energy expenditure, is known to reduce the risk of chronic diseases and premature mortality by 20–30% [3]. However, elevated body fat percentage fundamentally disrupts the efficiency of this energy expenditure, as individuals with higher adiposity incur a disproportionately greater metabolic cost for equivalent physical workloads [4,5], reflecting an underlying compromise in physiological efficiency.

High BF% is a growing global public health concern, with obesity affecting over 1.9 billion adults worldwide and 650 million classified as clinically obese [6]. In Saudi Arabia, the burden of elevated adiposity is particularly pronounced among females, with the prevalence of overweight and obesity reaching 35.5% compared to 31.8% in males [7]. Beyond its systemic metabolic consequences, elevated BF% exerts direct mechanical and functional impairments on the respiratory system [8]. From a physiological perspective, excess adiposity disrupts respiratory mechanics, skeletal muscle biology, and whole-body metabolism through three interrelated mechanisms. Excessive adipose tissue deposition around the thoracic cavity increases airway resistance, reduces pulmonary volumes, and elevates the mechanical load on respiratory muscles, collectively compromising maximal inspiratory and expiratory pressure generation [9,10]. These respiratory impairments further diminish cardiorespiratory fitness, which is itself strongly modulated by BF%, creating a compounding cycle of physiological inefficiency and elevated energy expenditure during exercise [10]. In addition, adipose tissue acts as an active endocrine organ, secreting adipokines (leptin, adiponectin, and resistin) and pro-inflammatory cytokines (TNF-α and IL-6) that sustain chronic low-grade inflammation [10,11,12,13]. This inflammatory milieu impairs muscle regeneration and promotes sarcopenic obesity [12], while simultaneously driving airway inflammation and bronchial hyperresponsiveness that amplify dyspnea perception [14,15].

Mechanically, obesity reduces lung and chest wall compliance, promotes airway closure, and increases resistive and elastic loads, thereby decreasing functional residual capacity and increasing airway resistance [11,15,16]. To compensate, individuals adopt a rapid, shallow breathing pattern that is energetically inefficient per unit of alveolar ventilation, substantially increasing respiratory muscle oxygen consumption [17].

At the muscular level, adiposity induces intramuscular fat infiltration, collagen deposition, fibro-adipogenic remodeling [18], and a metabolic shift toward glycolysis—all of which reduce contractile efficiency and oxidative capacity [10]. Centrally, leptin modulates respiratory drive and energy balance; early obesity-associated hyperleptinemia may transiently enhance minute ventilation and thermogenesis, partially offsetting increased mechanical load and preserving normocapnia [10,13]. In humans, increased intermuscular adiposity is linked to lower oxidative capacity and a metabolic shift toward glycolysis during exercise, reflecting decreased muscular efficiency. Although these findings are primarily derived from limb muscles, similar mechanisms are likely to affect respiratory muscles, thereby increasing the energetic cost of breathing [19]. Notably, the physiological effects of adiposity are not uniform and depend on factors such as sex, obesity status, age, and overall body composition. Recent evidence demonstrates that moderate fat mass may exert protective effects on certain tissues, such as bone, in non-obese individuals, whereas excessive adiposity—particularly in obese individuals—is associated with detrimental outcomes, including decline in tissue quality [20]. Together, these mechanical, inflammatory, and metabolic mechanisms underpin the observed relationships between adiposity, respiratory muscle strength, and energy expenditure.

While prior studies have independently examined the effects of BF% on respiratory muscle function and the relationship between adiposity and energy expenditure [21,22,23,24], these investigations have largely addressed these relationships in isolation. To date, no study has explored the mediating association of BF% in the pathway linking respiratory muscle strength to exercise energy expenditure. This represents a critical gap in the literature, as examining whether excess adiposity is statistically associated with the relationship between respiratory muscle strength and energy expenditure may help clarify how the physiological burden of obesity during physical activity is conceptualized. Elucidating this association is of particular clinical significance, as it may identify BF% as a modifiable target through which improvements in respiratory muscle function translate into meaningful reductions in the metabolic cost of exercise. Without examining these cross-sectional associations, the current understanding of the interplay between adiposity, respiratory mechanics, and energy metabolism remains incomplete, which may limit the development of targeted, evidence-informed strategies for individuals with elevated BF%.

## 2. Materials and Methods

### 2.1. Study Design and Population

A cross-sectional study was conducted involving 126 Saudi women aged 18–45 years. This study was conducted in accordance with the Strengthening the Reporting of Observational Studies in Epidemiology (STROBE) statement and the Guidelines for Reporting Mediation Analyses of Randomized Trials and Observational Studies [25,26]. Participants were recruited using a non-probability convenience sampling method via university campus announcements, social media platforms, and word-of-mouth referrals.

**Figure 1:**
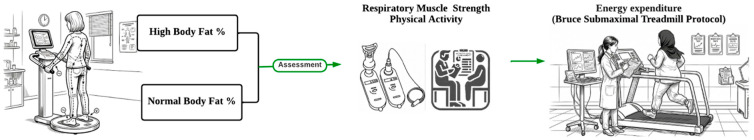
Flow diagram of the study protocol.

The inclusion criteria required that participants be women aged 18–45 years with BMI ≥ 18.5 kg/m^2^ and the ability to walk independently on a motorized treadmill. The exclusion criteria encompassed a history of cardiopulmonary, neurological, or musculoskeletal conditions, obstructive lung disease, use of medications known to affect cardiovascular function, current or recent nicotine use, and pregnancy. Participants were recruited from the general population; however, the sample predominantly consisted of young adults (mean age 21.71 ± 4.20 years; range: 18–41 years). Participants were stratified into two groups based on percentage of BF% cutoffs by sex and age in conjunction with BMI classification [27]. The high-body-fat group comprised women with a BF% exceeding 32% and a BMI between 25 and 29.9 kg/m^2^, whereas the normal-body-fat group included women with a BF% ranging from 21% to less than 32% and a BMI below 24.9 kg/m^2^. The participant flow and group allocation process are illustrated in Figure 1.

### 2.2. Ethical Considerations

This study was approved by the Internal Review Board Committee of Princess Nourah bint Abdulrahman University (IRB no. 24-0703) on 1 May 2024. Detailed explanations of the study protocol, procedures, and participant rights were provided to the participants prior to participation. Informed consent was obtained from all individual participants included in the study.

### 2.3. Sample Size Calculation

The cohort size was determined based on a previously reported study by Aldhahi et al. [28] on the effect of fat percentage on fitness. The calculations were performed using G*Power version 3.1 software (Heinrich-Heine-Universität Düsseldorf, Düsseldorf, Germany) to achieve 80% statistical power at a significance level of 0.05. A minimum sample size of 120 participants was determined to be sufficient to detect an effect size of 0.3. This calculation was based on a two-sided, two-sample *t*-test and linear regression to assess the primary variable of interest. Initially, a total of 127 participants were enrolled; however, one participant was subsequently withdrawn from the study due to incomplete data collection arising from failure to complete all required testing procedures, resulting in a final analytical sample of 126 participants.

### 2.4. Study Procedures

Participants were evaluated for inclusion criteria and screened for exercise suitability using the general health history questionnaire and Physical Activity Readiness Questionnaire plus (PARQ+). Anthropometric measurements were obtained before each participant underwent the modified Bruce protocol. All measurements were performed by trained personnel to ensure consistency and minimize inter-observer variability.

#### 2.4.1. Anthropometric Assessment

Height was measured to the nearest 0.1 cm using a calibrated wall-mounted stadiometer with participants standing barefoot and erect in the Frankfort plane. Body weight, BMI (kg/m^2^), fat-free mass percentage, and BF% were determined using bioelectrical impedance analysis with validated multifrequency devices (Seca mBCA 515, Hamburg, Germany), which measure impedance through the hands and feet while standing barefoot on the platform—a practical, non-invasive method validated against reference techniques, such as dual-energy X-ray absorptiometry [29].

Impedance was measured over 75 s with a current of 100 μA at a frequency range of 1 kHz to 1000 kHz and a capacity of 360 kg. Waist circumference was measured manually using a tape measure at the umbilicus, and physical activity level was used to estimate body composition. BMI was calculated as weight divided by height squared (kg/m^2^). Prior to testing, participants were advised to refrain from strenuous exercise and coffee consumption for 24 h prior to the assessment and to fast for at least 4 h to ensure accurate body composition measurements. All anthropometric measurements were performed by the same trained investigator to minimize inter-observer variability and ensure data consistency.

#### 2.4.2. International Physical Activity Questionnaire (IPAQ)

Using data from the short form 7-Day Physical Activity Recall questionnaire, all participants were asked about their weekly physical activity status [30]. The total amount of time they had spent exercising was also calculated. The IPAQ measures four levels of physical activity intensity [31]: walking, sitting, moderate-intensity activity, and vigorous-intensity activity. It also captures the participants’ recollection of their physical activity for the previous seven days. Cronbach’s alpha for the IPAQ was 0.76, indicating a high level of internal consistency reliability [32].

To determine METs per week, multiply the METs value provided (walking = 3.3, moderate activity = 4, and vigorous activity = 8) by the duration of the activity and then by the number of days the activity was performed [33]. To obtain the total METs of physical activity per week, we summed the METs attained in each group (walking, moderate activity, and vigorous activity) [30].

#### 2.4.3. Respiratory Muscle Strength

Respiratory muscle strength was assessed using MicroRPM (Puma PC, Micro Medical, v.2.2, Rochester, UK), a portable, non-invasive, mouth press with a rubber-edged mouthpiece and a small screen that displays test results digitally in cmH_2_O. Piezo-resistive pressure-sensing technology was used in the display. Maximal inspiratory pressure (MIP) and maximal expiratory pressure (MEP) were calculated using MicroRPM software (Version 2.2). The one-second average maximal pressure was used to determine the MIP and MEP values.

The participants were instructed to firmly close their lips around the flanged mouthpiece while holding the gauge with both hands. To perform the MEP maneuver, the participants were instructed to inhale as much as possible and then exhale as much as possible against the gauge’s resistance for more than 1 s. The participants in the MIP maneuver were instructed to exhale as much as possible (to residual volume) and then inhale as much as possible against the gauge’s resistance for a prolonged period. Each maneuver was repeated thrice, and the average between them was calculated [34].

#### 2.4.4. Bruce Submaximal Treadmill Protocol

**Figure 2:**
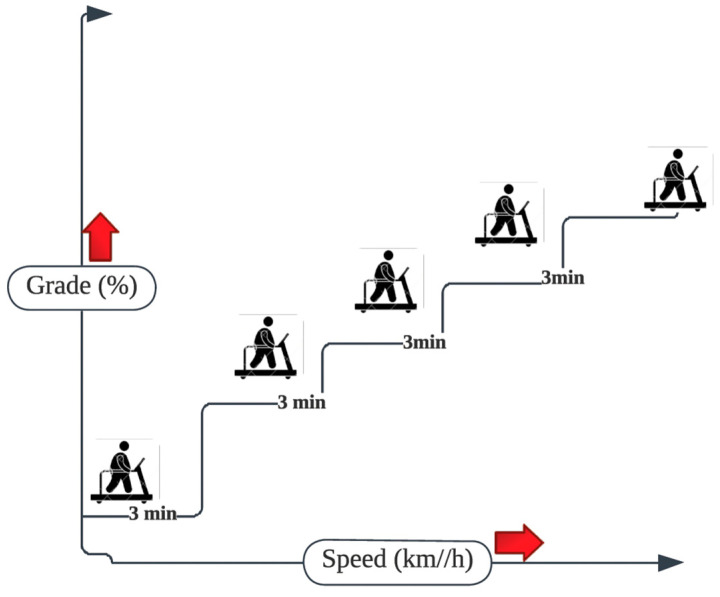
Illustration of the Bruce submaximal treadmill protocol. The arrows reflects stepwise increase.

This protocol describes a treadmill test that is multistage and used to measure cardiopulmonary fitness. The test was conducted in 3-min intervals until the participant reached 95% of their maximal heart rate, which is indicative of volitional exhaustion (Figure 2). A Polar heart rate monitor (Polar Electro Inc., Bethpage, NY, USA) was used to measure the participant’s heart rate simultaneously every two minutes to monitor the volitional exhaustion point and to ensure their safety. The Borg Rating of Perceived Effort [RPE] 6–20 [35], which measures the rate of perceived effort, and 95% of the age-predicted maximal heart rate were the criteria for terminating the motor-driven treadmill protocol. The test is often performed with maximal effort to anticipate optimal oxygen uptake, which is reflected by oxygen intake (VO_2peak_ [mL·kg^−1^·min^−1^]) and the length of exercise [28,36]. The participants were instructed to walk for 12 min on the treadmill with gradual increases in speed and grade. After a three-minute warm-up at one mph and zero grade, the speed and grade increased every three minutes by 2% until volitional exhaustion. Before the exercise, brachial blood pressure was manually measured at rest. During recovery, measurements were taken 1 min and 6 min post-exercise using a Polar heart rate monitor [37].

The VO_2peak_ was determined using the prediction equation, which has been validated for women [38]:

VO_2peak_ = 4.38 × (time) − 3.90

Energy expenditure, also referred to as caloric cost, is the amount of energy used in relation to the demands of metabolism. Energy expenditures are determined using the following operational formula:

(Energy expenditure = (Metabolic equivalents × 3.5 in mL·kg^−1^**·**min^−1^ × body  weight in kg)/(200 in kcal·mL^−1^).                  

where 200 kcal·mL^−1^ is 1000 mL·L^−1^ ÷ 5 kcal·L^−1^, unit kcal/min, and the Harris–Benedict equations formulas are used to provide a more tailored baseline metabolic rate than the generic “3.5 mL·kg^−1^**·**min^−1^” constant [39].

### 2.5. Statistical Analysis

Data analysis was performed using Stata software (version 17.0; StataCorp, College Station, TX, USA). Descriptive statistics are presented as means ± standard deviations for continuous variables and frequencies (percentages) for categorical variables. The Shapiro–Wilk test will assess normality of distributions. Between-group comparisons of participants with normal versus high body fat percentage were conducted using independent *t*-tests for normally distributed variables or Mann–Whitney U tests for non-parametric data. Chi-square tests were used to examine categorical variables. For continuous outcomes, Cohen’s d was calculated and interpreted as small (0.2), medium (0.5), and large (0.8) effects. For categorical variables, Cramér’s V was used, with values interpreted based on the strength of association (small, moderate, and large effects depending on degrees of freedom).

Spearman’s rho (ρ) correlation coefficients are used to evaluate bivariate associations between VO_2peak_, BF%, physical activity levels, respiratory muscle strength (MIP and MEP), and energy expenditure. Prior to regression modeling, multicollinearity was assessed using variance inflation factors (VIF < 10), and the assumptions of linearity, independence, homoscedasticity, and normality of residuals were verified through appropriate diagnostic plots and tests.

A hierarchical regression approach was used to evaluate the incremental association of variables entered in the following sequence: (1) body composition (fat percentage), (2) respiratory muscle strength parameters (MIP and MEP), (3) physical activity level (MET-minutes/week), and (4) energy expenditure. The change in R^2^ at each step determined the additional variance explained by each variable group.

To explore whether respiratory muscle strength mediates the relationship between BF% and energy expenditure, a mediation analysis was conducted using the procedure described by Baron and Kenny [40], accompanied by Jamovi’s Advanced Mediation Models 1.0.4 module (Jamovie, v. 1.6, 2020, The Jamovi Project, Sydney, Australia). Linear regression models were fitted to estimate the effects: (a) the total or indirect effect of MIP or MEP on energy expenditure, (b) the direct effect of MIP or MEP on fat% and (c) the effect of fat% on energy expenditure. The Sobel test was additionally performed to verify the significance of indirect effects. Statistical significance was set at *p* < 0.05 for all analyses.

## 3. Results

### 3.1. Participant Characteristics

The study included 126 participants, with 63 in the normal fat percentage group and 63 in the high fat percentage group. Table 1 presents the sociodemographic and physical characteristics of participants stratified by BF%. The two groups were similar in terms of age (mean difference was 1.7 years [95% CI [−3.15, −0.24], *p* = 0.24) and height (mean difference of 0.03; 95% CI [−2.23, 2.17], *p* = 0.76). Physical activity levels (METs/week) were not significantly different between the normal-BF group (95% CI [1230.48, 1996.82]) and the high-BF group (95% CI [1032.14, 1725.73]), *t*(124) = 0.91, *p* = 0.36, with a mean difference of 234.71 (95% CI [−277.00, 746.43]).

The majority of participants in both groups were undergraduate students, with minimal representation of postgraduate students (Table 1). However, occupation status differed significantly between groups (*p* = 0.015). Nearly all participants were single (98% in normal fat vs. 90% in high fat group), though this difference approached but did not reach statistical significance (*p* = 0.052).

Despite the substantial differences in body composition, both groups reported similar physical activity levels as measured by the IPAQ, with no significant differences in METs-minutes per week (*p* = 0.58). The distribution across activity categories was nearly identical, with approximately 46% classified as low activity, 38–40% as moderate, and 14–16% as high activity level in both groups (*p* = 0.96). The distribution of PA categories was similar across both study groups. A chi-square test of independence indicated no significant association between PA classification and groups (χ^2^ (2, N = 126) = 0.07, *p* = 0.964).

### 3.2. Respiratory Muscle Strength and Energy Expenditure Across the Group

A comparison between the normal and high-BF% groups revealed significant differences across all respiratory and metabolic parameters (Table 2). Participants with high BF% demonstrated substantially lower respiratory muscle strength than those with normal BF. MIP was significantly higher in normal-BF group (M = 87.04, SD = 14.08, 95% CI [83.49, 90.58]) compared to high-BF group (M = 64.17, SD = 13.15, 95% CI [60.86, 67.48]), *t*(124) = 9.43, *p* < 0.001, with a mean difference of 22.87 mmHg (95% CI [18.07, 27.68]). While MEP was approximately 31% lower in the high-BF group (95% CI [63.76, 71.36]) compared to the normal-BF group (95% CI [92.44, 102.32]) (both *p* < 0.001). These differences represented large effect sizes (Table 2). Thus, VO_2_ peak values were approximately 13% low (95% CI [31.94, 35.82]), with low METs observed in the high-BF group of approximately 14% compared to participants with normal BF percentage (95% CI [27.53, 31.12]) and showed moderate effect sizes (ES = 0.61 and 0.62, respectively). Energy expenditure showed an inverse pattern, with the high-BF group demonstrating approximately 26% higher energy expenditure (95% CI [10.36, 11.79]) during exercise compared to the normal-BF group (95% CI [8.23, 9.35], *p* < 0.001, ES = –1.26). However, as energy expenditure was calculated using an equation incorporating body weight, these high values may partially reflect the greater body mass of this group rather than solely physiological differences.

### 3.3. Association Between Respiratory Muscle Strength, Body Fat and Energy Expenditure

**Figure 3:**
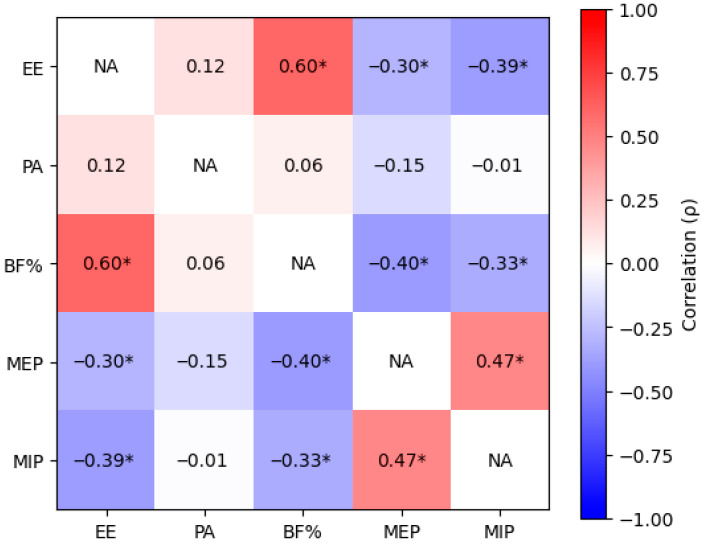
Heatmap of Spearman’s Correlation Matrix (ρ) illustrates the pairwise correlations among Energy Expenditure (EE), Physical Activity (PA), Fat percentage (Fat), Maximal Expiratory Pressure (MEP), and Maximal Inspiratory Pressure (MIP). Cells are color-coded using a diverging scale, where red shades indicate positive correlations and blue shades indicate negative correlations. The intensity of the color reflects the strength of the relationship, with darker shades representing stronger correlations and lighter shades indicating weaker associations. Diagonal cells are labeled “NA”, as self-correlations are not applicable. Statistically significant correlations (*p* < 0.05) are marked with an asterisk (*). Abbreviations: EE (kcal/min), Energy Expenditure; PA (METs), Physical Activity expressed as Metabolic Equivalents; body fat percentage (BF%); MEP (mmHg), Maximal Expiratory Pressure; MIP (mmHg), Maximal Inspiratory Pressure.

Figure 3 presents Spearman’s correlation coefficients examining the relationships between energy expenditure, physical activity, BF%, respiratory muscle strength, and cardiorespiratory fitness.

Energy expenditure was strongly positively correlated with BF% (ρ = 0.60, *p* < 0.05) but was moderately negatively correlated with both respiratory muscle strength measures (MIP: ρ = −0.390; MEP: ρ = −0.304, both *p* < 0.05). Physical activity levels (METs) showed no significant correlations with any measured parameters, indicating a disconnect between self-reported activity and objective physiological measures.

Hierarchical regression analysis (Table 3) revealed the incremental contribution of each variable group to energy expenditure variance explanation. The final model was statistically significant (F (4, 121) = 21.36, *p* < 0.001) and explained 41.40% of the variance in energy expenditure. Body fat percentage, entered in the first step, remained the most strongly associated variable (β = 0.078, *p* < 0.001), initially explaining 32.9% of the variance. The addition of maximal inspiratory pressure in step two increased the explained variance by 7.40%. The contribution of MEP was minimal (β = −0.006), and physical activity showed a negligible positive association (β = 0.00017). These findings indicate that b BF% and respiratory muscle strength are variables most strongly associated with energy expenditure during exercise, with BF% showing the strongest statistical association.

### 3.4. Mediating Analysis of Body Fat on the Relationship Between Respiratory Muscle and Energy Expenditure

Structural equation modeling was conducted to determine whether BF% mediates the relationship between MIP and energy expenditure. The mediation analysis revealed a significant indirect effect of MIP on energy expenditure through BF% (indirect effect = −0.016, SE = 0.007, z = −2.13, *p* = 0.033, 95% CI: −0.031 to −0.001).

**Figure 4:**
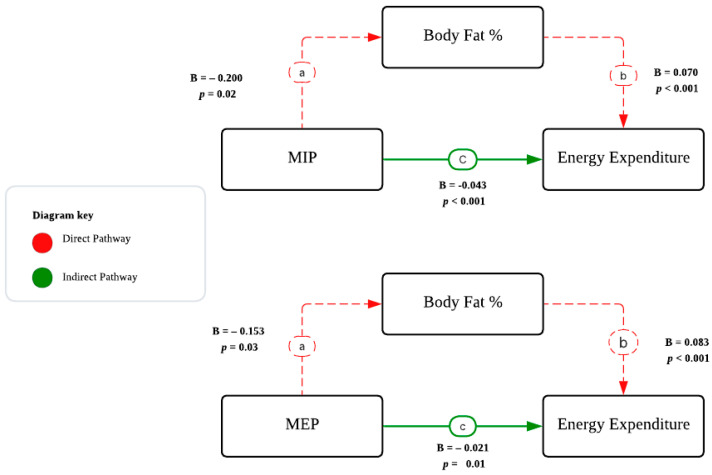
Statistical mediation model illustrating the associations among muscle strength, energy expenditure and body fat percentage.

The Baron and Kenny approach confirmed partial mediation (Figure 4). MIP was significantly associated with BF% (B = −0.200, *p* = 0.026), body fat was significantly associated with energy expenditure (B = 0.070, *p* < 0.001), and the effect of MIP on energy expenditure remained significant after controlling for body fat (B = −0.043, *p* < 0.001).

The relative indirect effect (RID) indicated that approximately 27% of the total effect of MIP on energy expenditure was associated with BF%. The ratio of indirect to direct effects (RID = 0.365) suggests that the mediated effect was about 0.4 times as large as the direct effect. These findings indicate that weaker respiratory muscle strength is associated with high energy expenditure both directly and indirectly via its statistical association with BF%, with body fat consistent with a partial mediator in this cross-sectional model.

A similar mediation analysis was conducted for maximal expiratory pressure (MEP). BF% was statistically consistent with a mediating role in the relationship between MEP and energy expenditure (indirect effect = −0.013, SE = 0.006, z = −2.11, *p* = 0.035, 95% CI: −0.024 to −0.001). The mediation analysis showed that MEP was significantly associated with BF% (B = −0.153, *p* = 0.03), body fat was associated with energy expenditure (B = 0.083, *p* < 0.001), and MEP maintained a significant effect on energy expenditure after controlling for body fat (B = −0.021, *p* = 0.01), indicating partial mediation. Notably, the relative indirect effect revealed that 38% of MEP’s total effect on energy expenditure was mediated through BF%, compared to 27% for MIP. The ratio of indirect to direct effects (RID = 0.606) indicates that the indirect association through body fat was approximately 0.6 times the magnitude of the direct association.

## 4. Discussion

This study investigated the complex interplay between adiposity, respiratory muscle performance, and metabolic demand during exercise. Our primary objective was to examine the extent to which BF% statistically mediated the cross-sectional association between respiratory muscle strength and exercise energy expenditure. The results demonstrate that individuals with high exhibit significantly diminished maximal inspiratory and expiratory pressures, alongside lower VO_2peak_ and METs. Conversely, these individuals showed significantly high metabolic costs during exercise. Body fat % showed the strongest statistical association with energy expenditure and was consistent with a partial mediation model in the relationship between respiratory muscle strength and metabolic cost—accounting for 27% and 38% of the effect for MIP and MEP, respectively. These findings indicate statistically significant associations between adiposity, respiratory muscle strength, and metabolic cost, without implying causal directionality.

The lower respiratory pressures observed in the high-BF group are partly attributable to the mechanical effects of excess adiposity on respiratory mechanics. Excess adipose tissue in the thoracic and abdominal compartments imposes a mechanical load that reduces chest wall compliance and restricts diaphragmatic excursion [10,41]. This mechanical impedance places the diaphragm at a functional disadvantage, necessitating increased neural drive to overcome elastic loads [24,42]. However, this mechanical explanation alone is unlikely to fully account for the observed reductions in MIP and MEP. Respiratory muscle performance in individuals with higher adiposity is likely to be multifactorial. In addition to mechanical constraints, factors such as physical deconditioning, altered neuromuscular activation, and potential systemic metabolic or inflammatory influences may contribute to impaired respiratory muscle function [24]. It likely represents a combination of mechanical disadvantage and systemic deconditioning. Therefore, the lower MIP and MEP values observed in the high-BF% group should be interpreted as reflecting a combination of mechanical loading and broader physiological influences, rather than a single underlying mechanism [10].

Participants in the high-BF group had higher energy expenditure despite lower METs, which points to a significant reduction in mechanical efficiency. The metabolic cost of locomotion is elevated in individuals with higher adiposity because of the increased oxygen cost required to move larger limb masses and overcome internal resistance [5,42]. The discrepancy between self-reported physical activity and physiological measures further underscores that the higher energy expenditure in the high group is not a byproduct of higher fitness, but rather a marker of physiological inefficiency and inadequate ventilatory reserves [43]. Although the high-body-fat group exhibited higher absolute energy expenditure, this finding should be interpreted with caution, as the calculation inherently includes body weight and may therefore overestimate differences between groups. Importantly, METs were derived from VO_2_ estimates obtained during the treadmill protocol, reflecting physiological responses to exercise rather than purely mathematical scaling. Therefore, the observed differences are unlikely to be explained solely by the calculation method and may reflect a combination of physiological and methodological factors.

The moderate negative correlations between MIP/MEP and energy expenditure suggest that enhanced respiratory muscle strength is associated with metabolic efficiency. Stronger respiratory muscles may facilitate improved ventilatory efficiency and decline in oxygen cost of breathing, thereby delaying the onset of respiratory muscle fatigue [43,44]. The stronger association observed for MIP compared to MEP may be due to the fact that the work of inspiration is more critically hindered by the mechanical constraints of obesity, such as the cranial displacement of the diaphragm and increased intra-abdominal pressure [10,45].

Regression analysis quantified BF% as most strongly associated variable, explaining 32% of the variance in energy expenditure. However, the inclusion of MIP significantly improved the model’s explanatory power (increase of 7.3–8.3%), indicating that inspiratory strength provides unique information regarding metabolic cost that is not captured by adiposity alone. The minimal contribution of self-reported physical activity suggests that metabolic cost during exercise is more closely tied to objective physiological and anthropometric parameters than subjective activity levels.

The mediation analysis revealed that BF% was significantly associated with both respiratory muscle strength and energy expenditure, consistent with a mediating role in the analytical model. However, this analysis did not establish that body fat mechanistically explained these relationships. The observed associations may reflect multiple underlying processes. The significant indirect effects identified for both MIP and MEP confirm a partial mediation model. This suggests that while respiratory muscle strength is independently associated with energy expenditure, a substantial portion of this relationship is statistically associated with the structural and metabolic consequences of adiposity. The remaining direct effect suggests that respiratory muscle performance is associated with exercise efficiency independently of the participant’s body composition.

We suggest a possible association in which low respiratory muscle strength may be associated with decline in exercise tolerance and ventilatory capacity, potentially leading to a sedentary lifestyle and subsequent accumulation of body fat [41]. Higher adiposity is associated with greater metabolic cost of movement through mechanical loading. However, given the cross-sectional nature of this analysis, we must also acknowledge the reverse pathway in which obesity-related mechanical loads directly impair respiratory muscle performance [10]. Thus, a possible interpretation of these findings is that lower respiratory muscle strength, adiposity, and energy expenditure are interrelated; however, the direction of these relationships cannot be determined using this cross-sectional design. Alternative explanations are equally plausible, including the possibility that adiposity contributes to low respiratory muscle performance through mechanical restriction or that both variables are influenced by shared factors, such as physical deconditioning, metabolic status, or lifestyle behaviors.

The relative indirect association values of MIP and MEP indicate differential patterns in the statistical mediation model. The relationship between MEP and energy expenditure is more strongly mediated by BF, whereas MIP retains a more substantial direct association with metabolic cost. This suggests that inspiratory strength may be more independently associated with exercise efficiency in women.

These associations are consistent with the potential relevance of respiratory muscle training in obesity management protocols as improved strength is associated with reduced burden and metabolic cost of exercise [10,44]. Furthermore, our findings reinforce the clinical necessity of assessing BF% over BMI alone, as it more accurately reflects the mechanical and metabolic constraints on the respiratory system [45,46]. In a cross-sectional study of 833 Slovak young adults, the prevalence of normal-weight obesity was 31.5% in women, with affected individuals displaying significantly elevated visceral fat area and reduced lean body mass [47]. Therefore, individuals classified as “normal weight” by BMI may nonetheless harbor the visceral adiposity and reduced lean mass that impair respiratory muscle function and elevate the energetic cost of exercise. Normal weight obesity thus reinforces the rationale for prioritizing BF% over BMI and underscores that metabolic and ventilatory risk may be substantially underestimated when screening by BMI alone. Additionally, these findings highlight that individuals with obesity have decrease in breathing reserve at peak exercise, as the heightened ventilatory demand approaches their limited ventilatory capacity due to the increased metabolic cost of moving heavier limbs [48].

Despite the significant findings regarding the mediating role of body fat, several limitations must be acknowledged. Given the cross-sectional and observational design of this study, causal inferences between respiratory muscle strength, adiposity, and energy expenditure cannot be established. The temporal sequence of these associations remains unclear, and the possibility of bidirectional relationships cannot be excluded [49]. Accordingly, all findings should be interpreted as statistical associations rather than evidence of causal pathways or mechanistic relationships. While bioelectrical impedance analysis is a practical and accessible tool, it is not the gold standard for body composition assessment [50]. Additionally, energy expenditure and aerobic capacity were assessed using indirect (estimated) measures of VO_2_ rather than direct metabolic cart analysis, which may reduce measurement precision. BIA may underestimate fat mass in individuals with high adiposity, and, unlike dual-energy X-ray absorptiometry, it cannot distinguish between visceral and subcutaneous fat depots [51]. This is a critical distinction, as visceral fat in the thoracic and abdominal regions has a more profound restrictive effect on diaphragmatic excursion than subcutaneous fat. Another limitation is the reliance on self-reported physical activity, which is prone to recall bias. Although physical activity contributed minimally to the regression model, this finding must be interpreted cautiously, as differential misreporting between adiposity groups may have attenuated the true association.

This study utilized a convenience sample of female participants, which may have introduced selection bias as participants were likely motivated individuals. Consequently, the findings may not be generalizable to the broader population, including men or older adults, particularly as gender differences in respiratory physiology and fat distribution are well-documented [52].

## 5. Future Research Directions

The cross-sectional nature of this study precluded the establishment of a definitive causal relationship. Consequently, longitudinal and randomized controlled trials are required to determine whether improvements in respiratory muscle strength are associated with enhanced metabolic efficiency or whether weight reduction is the primary variable associated with improved ventilatory mechanics [21].

Furthermore, while this study utilized total BF%, future research should incorporate advanced imaging techniques, such as dual-energy X-ray absorptiometry or magnetic resonance imaging, to differentiate between visceral and subcutaneous adipose tissue [53]. Visceral fat is more metabolically active and has been more strongly linked to impaired lung function and restricted diaphragmatic excursion than subcutaneous fat [54,55]. Investigating these specific fat depots would clarify the precise mechanical vs. metabolic load imposed on the respiratory system. Additionally, the role of systemic inflammation and autonomic regulation warrants further investigation. Obesity is characterized by chronic low-grade inflammation and sympathovagal imbalance, which can impair respiratory muscle performance and increase cardiovascular risk [54].

Finally, future cohorts should include male participants and broader age ranges (including adolescents and older adults) to investigate sex-specific and age-related differences in fat distribution and its statistical association with respiratory function in mediation models [21,48]. Integrating direct metabolic cart measurements instead of submaximal predictions would further enhance the precision of metabolic demand assessment in these populations [53].

## 6. Conclusions

Body fat percentage serves as a critical mediator in the relationship between respiratory muscle strength and exercise energy expenditure. Although adiposity is the primary driver of increased metabolic demand, respiratory muscle strength—particularly inspiratory strength—remains an independent associated factor with metabolic efficiency. Interventions targeting both body composition and respiratory performance may be the most effective strategy for optimizing exercise tolerance and reducing metabolic burden in women. However, these findings are associative and require longitudinal validation. Ultimately, these results underscore the importance of integrating targeted inspiratory muscle training into weight management programs to mitigate ventilatory limitations and enhance overall exercise tolerance. Implementing such multifaceted approaches may specifically address the heightened mechanical load and potential metabolic-reflex-driven muscle fatigue that currently restricts the aerobic capacity of women with higher body fat.

## Figures and Tables

**Table 1:** Demographic, Anthropometric, and Physical Activity Characteristics According to Body Fat Percentage Groups.

Variable	Body Fat Percentage (BF%)		*p*-Value	Effect Size ^¥^
	Normal n = 63 Mean ± SD	High n = 63 Mean ± SD		
Age (years)	20.85 ± 2.36	22.55 ± 5.34	0.24	0.41
Weight (kg)	51.94 ± 6.49	76.26 ± 12.19	<0.001	2.48
Height (cm)	159.34 ± 7.13	159.38 ± 5.17	0.76	0.005
BMI (kg/m^2^)	20.63 ± 2.27	30.13 ± 5.06	<0.001	2.41
Occupation Status, (n)%				
Not employed	0 (0)	1 (1.59)	0.01	0.25
Student	60 (95.24)	49 (77.78)		
Employed	3 (4.76)	13 (20.63)		
Educational Level, (n)%				0.04
High school or less	0 (0)	0 (0)	0.64	
Undergraduate (Associate/bachelor’s degree)	61 (96.83)	60 (95.24)		
Postgraduate (Master’s/Doctorate)	2 (3.17)	3 (4.76)		
Marital Status, n (%)				
Single	62 (98.41)	57 (090.48)	0.05	0.17
Married	1 (1.59)	6 (9.52)		
Divorced	0 (0)	0 (0)		
Widowed	0 (0)	0 (0)		
Physical activity IPAQ (METs/week)	1613.65 ± 191.68	1378.93 ± 173.48	0.58	0.16
Physical activity level				
Low, n (%)	29 (46.03)	29 (46.03)	0.96	0.02
Moderate, n (%)	25 (39.68)	24 (38.10)		
High, n (%)	9 (14.29)	10 (15.87)		

Mann–Whitney U test used for continuous variables. ^¥^ Cohen’s d for continuous variables and Cramér’s V for categorical variables. Abbreviations: BMI: Body Mass Index; IPAQ: International Physical Activity Questionnaire BMI: Body Mass Index.

**Table 2:** Comparison of Respiratory Muscle Strength and Energy Expenditure and Cardiorespiratory Fitness Parameters According to BF% Classification.

Variable ^†^	Body Fat Percentage (BF)		*p*-Value	Cohen’s d ES
	Normal n = 63 Mean ± SD	High n = 63 Mean ± SD		
Maximal Respiratory Pressures				
MIP, cmH_2_O	87.03 ± 14.07	64.16 ± 13.14	<0.001	1.67
MEP, cmH_2_O	97.37 ± 19.62	67.56 ± 15.09	<0.001	1.70
VO_2peak_, mL·kg^−1^**·**min^−1^	33.88 ± 7.68	29.32 ± 7.11	0.0008	0.61
Energy expenditure, kcal/min	8.78 ± 2.23	11.07 ± 2.84	<0.001	–1.26

^†^ Independent-samples *t*-test. Abbreviations: Peak Oxygen Consumption (VO_2peak_); Metabolic Equivalent (METs); Maximal Inspiratory Pressure (MIP in cmH_2_O); Maximal Expiratory Pressure (MEP in cmH_2_O); Effect Size (ES).

**Table 3:** Hierarchical regression analysis for the Energy expenditure as dependent variable.

Dependent	Predictors	Coefficients				f^2^	R^2^	ΔR^2^
		β	SE	95% CI				
				Lower	Upper			
Energy Expenditure (EE)	Constant	11.978	1.015	9.960	13.988		-	-
	BF%	0.078	0.010	0.056	0.099	0.470	0.329	-
	MIP	−0.040	0.012	−0.065	−0.015	0.121	0.403	0.074
	MEP	−0.006	0.009	−0.025	0.012	0.053	0.406	0.003
	PA (METs)	0.00017	0.0001	−0.00009	0.0004	0.130	0.413	0.007
F (4, 121)		21.36						
*p*-value		<0.001						
R^2^		0.414						

Abbreviations: β: standardized beta, SE: Standard errors. Abbreviations: Energy Expenditure (EE in kcal/min); Physical Activity (PA), expressed as Metabolic Equivalents (METs); Body Fat percentage (BF %); Maximal Expiratory Pressure (MEP in mmHg); Maximal Inspiratory Pressure (MIP in mmHg).

## Data Availability

The identified datasets analyzed during the current study are available from the corresponding author on reasonable request.

## Abbreviations

The following abbreviations are used in this manuscript:

BF%	Body Fat percentage
BMI	Body Mass Index
EE	Energy Expenditure
METs	Metabolic Equivalents
MIP	Maximal Inspiratory Pressure
MEP	Maximal Expiratory Pressure
PA	Physical Activity
RPE	Rating of Perceived Exertion
STROBE	Strengthening the Reporting of Observational Studies in Epidemiology
RMR	Resting Metabolic Rate
VO_2_peak	Peak Oxygen Consumption (mL·kg^−1^**·**min^−1^)
